# Factors related to work ability and well-being among women on sick leave due to long-term pain in the neck/shoulders and/or back: a cross-sectional study

**DOI:** 10.1186/s12889-018-5580-9

**Published:** 2018-05-30

**Authors:** Mamunur Rashid, Marja-Leena Kristofferzon, Marina Heiden, Annika Nilsson

**Affiliations:** 1 Centre for Musculoskeletal Research, Department of Occupational and Public Health Sciences, Faculty of Health and Occupational Studies, University of Gävle, SE-80176 Gävle, Sweden; 2 Department of Health and Caring Sciences, Faculty of Health and Occupational Studies, University of Gävle, Gävle, Sweden; 3 Department of Public Health and Caring Sciences, Faculty of Medicine, Uppsala University, Uppsala, Sweden

**Keywords:** Musculoskeletal pain, Sickness absence, Prognostic factors, Ability to work, Quality of life, Women

## Abstract

**Background:**

Musculoskeletal pain is one of the leading causes of sick leave, especially among women, in Western countries. The aim of the present study was to identify factors associated with work ability and well-being, respectively, among women on sick leave due to long-term pain in the neck/shoulders and/or back.

**Methods:**

A cross-sectional study with a correlational design was conducted on women who were sick-listed due to long-term pain in the neck/shoulders and/or back. A total of 208 participants responded to a survey comprising eight instruments: Multidimensional Pain Inventory scale, General Self-Efficacy scale, Sense of Coherence scale, Coping Strategies Questionnaire, Demand-Control-Support Questionnaire, Hospital Anxiety and Depression Scale, Work Ability Index and Life Satisfaction questionnaire. Multiple linear regression analyses were performed to identify factors associated with work ability and well-being, respectively.

**Results:**

Women who more strongly believed they would return to the same work had greater work ability (β = 0.39, *p* < 0.001), whereas women with higher pain intensity (β = − 0.30, *p* < 0.001) and higher job strain (β = − 0.12, *p* < 0.05) had lower work ability. Women with higher self-efficacy rated greater well-being (β = 0.14, *p* < 0.05). As the women’s scores for depression increased, their well-being decreased by 48%, which was statistically significant (*p* < 0.001). The regression models for work ability and well-being were significant (*p* < 0.001), and their adjusted R- square values were 48% and 59%, respectively.

**Conclusions:**

The study suggests that the factors *beliefs to be back at the same work*, *pain intensity* and *job strain* are predictive of work ability. Moreover, the factors *self-efficacy* and *depression* seem to be predictive of well-being. The findings highlight factors that should be considered by health care professionals and policy-makers to guide attempts to reduce sick leave.

## Background

Sick leave due to long-term Musculoskeletal Pain (MSP) is considered an increasing public health problem in Western countries [1, 2]. The social economic costs of sick leave are immense owing to workers’ compensation, medical expenses and productivity loss [3, 4]. Long-term pain in the neck, shoulders and back is one of the leading causes of reduced work ability and well-being among workers [5]. Previous findings suggest that women have a higher prevalence of neck/shoulders and low back pain and are more likely to be on sick leave than men [2, 6, 7].

The concept of work ability comprises the physical, psychological and social capability of a worker, which interact with the workers’ health condition, physical and mental resources and work demands [8, 9]. In this study, work ability reflects the individual’s perceived current and near future ability to perform work with respect to work demands, health and mental resources [10]. In general, well-being reflects the meaningfulness of life with regard to physical, mental and social dimensions [11], and in this study, well-being is assessed through 10 dimensions of life satisfaction with regard to daily living using a self-report checklist (LiSat- 11) [12]. Owing to the importance of work ability and well-being in the working population [2, 13–16], it is essential to understand which factors are important for work ability and well-being in this population. Previous systematic reviews have attempted to identify work-related and individual factors of importance for work ability and well-being, but they have done so in the general working population or among people with MSP. For example, van den Berg et al. [17] found that older age, obesity, poor musculoskeletal capacity, high mental work demands, poor physical work environment, high physical work load, lack of autonomy and lack of physical activity have a negative effect on work ability among people with long-term MSP. Another review by Hoogendoorn et al. [18] presented strong evidence indicating that low social support at work and low job satisfaction are related to reduced well-being among back pain patients. It is possible that the factors of importance for work ability and well-being among people who are sick-listed are different owing to the severity of their conditions [5, 8, 19, 20].

A recent systematic review showed that recovery beliefs, health and work capacity were important for return to work among people on sick leave due to long-term pain in the neck or back [16]. The authors concluded, however, that more studies were needed to confirm the results. Generating more knowledge about what is needed to improve work ability and well-being, and consequently to facilitate return to work in this population would benefit individuals as well as society. However, previous studies have primarily focused on both women and men with sub-acute or chronic pain who were working or on part-time sick leave [19–24]. As studies have shown that work tasks tend to differ between men and women, even in the same line of work [25, 26], it is possible that factors influencing work ability also differs between genders. Therefore, they should be investigated separately. Thus, the aim of the present study was to identify factors associated with work ability and well-being, respectively, among women on sick leave due to long-term pain in the neck/shoulders and/or back.

## Methods

### Study design

The study was cross-sectional and used a correlational design.

### Sample

In spring 2016, a total of 600 women from central and northern Sweden were invited by the Swedish Social Insurance Agency (SIA) to take part in the study. They were selected on the basis of their medical certificate, issued by their primary health care or hospital physician. Women were included in the study if they met the following criteria: 18 to 65 years of age; ≥ 50% sick leave from service (i.e., they could do part-time work); duration of sick leave ≥1 month due to pain in the neck/shoulders (ICD code: M53.1, M54.2, M54.9, M75.8, M75.9 and M79.1) and/or back (ICD code: M54.4, M54.5, M54.9 and M79.1) for ≥3 months. They should also be able to read, write and understand Swedish. The exclusion criteria were: rheumatoid arthritis, multiple sclerosis, stroke, cancer, Parkinson, bipolar disease, schizophrenia and pregnancy. An information letter and questionnaires were sent to the women; two reminders were sent out. Of the 600 women, 275 responded to the questionnaires, giving a response rate of 46%. Of them, 67 were excluded based on exclusion criteria, thus 208 were included in the analyses. Because the Swedish Social Insurance Agency invited the women to take part in the study, the authors had no access to non-respondents’ data. For this reason, the non-response analysis cannot be performed.

### Data collection

Self-administered questionnaires based on eight instruments and a set of demographic variables such as age, education, country of birth, cohabitation, working conditions, economic situation, physical activity and beliefs to be back at the same work were sent to the women. In addition, a pain figure was included to collect information on the location of pain on the body [27]. Subscales from six instruments were treated as independent variables and two were used as outcome variables. A description of each instrument is provided below.

### Independent variables

#### Pain intensity

To assess pain intensity, the Multidimensional Pain Inventory (MPI- S) [28] was used. The psychosocial section (part 1) consists of 28 items forming five sub-scales. In the present study, we analyzed only one sub-scale: pain intensity, which consists of 3 items, where responses are made on a 7-point Likert rating scale (0 = no pain; 6 = extreme pain), and where higher scores indicate higher pain intensity. The internal consistency of the scale, measured using Cronbach’s α, was 0.76.

#### Self-efficacy

To assess self-efficacy, the General Self-Efficacy (GSE) [29] scale was used. It consists of 10 items that respondents rated on a 4-point Likert scale (1 = not true; 4 = completely true). Total scores ranged from 10 to 40 points, higher values indicating greater general self-efficacy. The internal consistency of the scale, measured using Cronbach’s α, was 0.92.

#### Sense of coherence

Sense of coherence (SOC) is part of the salutogenic approach of health that focuses on one’s ability to identify resources for health and well-being. SOC is the capability to manage whatever the situation demands in life to perceive life as comprehensible, manageable and meaningful [30]. The SOC scale [30, 31], short version, was used to assess sense of coherence. The scale consists of 13 items, with total scores ranging from 7 to 91 points. The respondents rated items on a 7-point scale (1 = never; 7 = very often), where higher scores represent greater SOC. The internal consistency of the scale, measured using Cronbach’s α, was 0.84.

#### Coping strategies

Coping strategies were assessed using the Swedish version of the Coping Strategies Questionnaire (CSQ) [32], which consists of 50 individual items forming 8 sub-scales. In the present study, we used only three of the subscales: *divert attention, ignore sensation, and increase behavioral activities*. Each item was rated on a 7-point Likert scale ranging from 0 = never to 6 = always, higher values representing more frequent use of the coping strategy. The internal consistency values for the sub-scales, measured using Cronbach’s α, were 0.87, 0.86 and 0.86, respectively.

#### Job strain and support at work

Job strain was assessed using the Demand Control Support Questionnaire (DCSQ) [33] which consists of 17 items forming four subscales: psychosocial demands, skills discretion, decision authority and support at work. For each item, responses were made on a 4-point Likert scale ranging from 1 (strongly agree) to 4 (strongly disagree). First, we constructed an index for each of the three scales: psychological demands, skills discretion and decision authority. Skills discretion and decision authority were then merged into one scale called decision latitude [34]. Afterwards, a job strain score was created by calculating the ratio between psychological demands and decision latitude, where higher values represent higher job strain [33]. For the subscale support at work, the values for the six items were summed to a total score, where higher values represent greater perception of support at work. The internal consistency of the scale, measured using Cronbach’s α, was 0.57 for job strain and 0.51 for support at work.

#### Anxiety and depression

Anxiety and depression were assessed using the Hospital Anxiety and Depression Scale (HADS) [35, 36] which consists of 14 items forming two scales: anxiety (7 items) and depression (7 items). Respondents rated each item on a 4-point Likert scale, where higher values indicate greater anxiety or depression. For each scale, a total score was calculated that ranged from 0 to 28 points. The internal consistency values for the scales, measured using Cronbach’s α, were 0.90 and 0.91, respectively.

Two additional factors were assessed using a single item each. Beliefs to be back at the same work were assessed using the question: Do you believe you will return to the same work within 6 months? These beliefs were assessed on a 10-point scale (from 1 = highly unlikely to 10 = highly likely). Physical activity was assessed using the question: How often do you exercise regularly for at least 30 min, e.g., walking, jogging, swimming, cycling or working in the garden? The four response alternatives were: 0 days/week, 1–3 days/week, 4–5 days/week, 6–7 days/week.

### Outcome measures

#### Work ability

Work ability was assessed using the Work Ability Index (WAI) [10, 37], which consists of 7 items. For each item, a single score was obtained, and the total WAI score was calculated by summing all single-item scores; the total score ranged from 7 to 49 points. Lower scores indicate lower work ability. The internal consistency of the scale, measured using Cronbach’s α, was 0.78.

#### Well-being

Well-being was measured using the Life Satisfaction questionnaire (LiSat- 11) [12, 38], which consists of 11 items. Each item was rated on a 6-point ordinal scale ranging from 1 = very dissatisfied to 6 = very satisfied, where high scores reflect greater life satisfaction. The items were averaged to produce the index of well-being. The internal consistency of the scale, measured using Cronbach’s α, was 0.86.

### Potential confounders

Age and economic situation were considered potential confounders in the analysis. They were chosen because they were found to be important confounders in a systematic review of factors important for return-to-work among people with long-term pain in neck or back [16]. Age was measured on a continuous scale, and economic situation was assessed on a 5-point scale (from 1 = very dissatisfied to 5 = very satisfied).

### Statistical analysis

All data analyses were performed using the statistical software IBM SPSS, version 22. Descriptive statistics of demographic variables are presented as proportions, means and standard deviations. Scatterplots showed that all variables were normally distributed and that there were no outliers in the data. Prior to the regression analyses, multi-collinearity diagnostics using variance inflation factor (VIF) were applied, where all VIF values were less than 3.9, indicating no problem with multi-collinearity between independent variables in the models [39]. Bivariate correlations between independent variables were also computed [40]. To determine the association between the independent variables and work ability and well-being, respectively, multiple linear regression analyses were performed separately for each of the outcomes. The analyses were performed with and without adjustment for age and economic situation, as data on 208 subjects gave sufficient statistical power to allow inclusion of all independent variables and confounders in the models simultaneously. The level of significance was set at *p* < 0.05.

## Results

Table 1 describes the participants’ demographic characteristics. The mean age was around 50 years (range 23–64 years). Ninety-six percent of the women were born in Sweden and 76% lived with a partner. Half of the women had upper secondary education, and 65% were satisfied with their economic situation. More than two-thirds of the women (70%) had blue-collar work and 30% white-collar. A total of 68% of the women experienced neck/shoulder pain, 71% back pain, and 43% had pain in both areas.

**Table 1:** Demographic characteristics of the participants (*n* = 201–208)

Variables	Frequency (%)
Age (years) (M, SD)	49.63 ± 9.71
Country of birth	
Sweden	200 (96)
Others	8 (4)
Cohabitation	
Living with partner	158 (76)
Living alone	39 (19)
Living apart	11 (5)
Education	
Elementary	42 (20)
Upper secondary	104 (50)
University	53 (26)
Others	9 (4)
Types of work	
Blue-collar	145 (70)
White-collar	63 (30)
^a^Working life prior to SL (years) (M, SD)	30.15 ± 10.75
Economic situation	
Very dissatisfied	25 (12)
Dissatisfied	43 (21)
Acceptable	88 (42)
Good	38 (18)
Very good	11 (5)
Pain area	
Neck/shoulders	142 (68)
Back	148 (71)
Neck/shoulders and back	89 (43)
Pain duration (months) (M, SD)	83.63 ± 99.64
Physical activity	
0 day/week	26 (13)
1–3 days/week	81 (39)
4–5 days/week	57 (27)
6–7 days/week	41 (20)
Beliefs to be back at the same work (1–10 scale) (M, SD)	6.56 ± 3.73

^a^Total working years before being sick-listed

*M* Mean and *SD* Standard deviation, *SL* Sick leave

Bivariate correlation coefficients between the independent variables were smaller than 0.55 (Table 2). Table 3 presents the associations between the independent variables and work ability. The results showed that women with higher pain intensity (β = - 0.30, *p* < 0.001) and higher job strain (β = − 0.12, *p* < 0.05) had lower work ability, whereas women who believed more strongly that they would return to the same work within 6 months had greater work ability (β = 0.39, *p* < 0.001). The regression model was significant (*p* < 0.001), and the independent variables explained 48% of the variance in work ability.

**Table 2:** Bivariate Pearson’s correlation coefficients between independent variables included in the regression analyses (*n* = 196–208)

Variables	1	2	3	4	5	6	7	8	9	10	11	12	13	14
1. Age	1													
2. Economic situation	0.02	1												
3. Pain intensity	0.14	0.07	1											
4. Self-efficacy	0.01	0.03	0.23^d^	1										
5. Sense of coherence	0.09	0.01	0.32^d^	− 0.06	1									
6. Divert attention	−0.06	− 0.02	− 0.12	0.30	0.41	1								
7. Ignore sensation	0.01	−0.10	−0.04	0.01	0.15	0.23^d^	1							
8. IBA^a^	0.02	− 0.10	0.11	0.04	0.01	0.43^d^	0.54^d^	1						
9. Job strain	0.03	−0.01	−0.08	0.01	0.13	−0.13	−0.13	0.10	1					
10. Support at work	−0.01	−0.06	−0.10	− 0.12	0.13	− 0.27^d^	−0.37^d^	− 0.01	−0.03	1				
11. Physical activity	−0.03	− 0.07	0.03	0.11	−0.07	0.01	0.01	0.04	−0.01	0.16^c^	1			
12. BBSW^b^	−0.15^c^	− 0.19^d^	0.23^d^	− 0.19^d^	−0.40^d^	0.11	−0.18^a^	− 0.23^d^	−0.06	− 0.02	−0.07	1		
13. Anxiety	−0.02	0.04	−0.26^d^	0.16^a^	0.25^d^	−0.47^d^	−0.55^d^	0.18	−0.01	0.07	0.14	0.32^d^	1	
14. Depression	−0.02	0.07	−0.32	0.13	0.35^d^	−0.51^d^	−0.13	− 0.12	−0.12	− 0.06	0.15	0.23^d^	0.46^d^	1

^a^*IBA* Increase behavioral activities

^b^*BBSW* Beliefs to be back at the same work

^c^Correlation is significant at the 0.05 level

^d^Correlation is significant at the 0.01 level

**Table 3:** Multiple linear regression analyses between the independent variables and work ability

Variables	Work ability (*n* = 207)					
	Unadjusted analysis			Adjusted analysis		
	β	SE	*p*-value	β	SE	*p*-value
Pain intensity	**− 0.32**	**0.46**	**0.001**	**−0.30**	**0.47**	**0.001**
Self-efficacy	0.12	0.10	0.08	0.11	0.10	0.11
Sense of coherence	−0.11	0.05	0.20	−0.10	0.05	0.31
Divert attention	0.04	0.10	0.52	0.02	0.11	0.78
Ignore sensation	−0.11	0.09	0.11	−0.12	0.09	0.10
Increase behavioral activities	0.04	0.11	0.61	0.06	0.11	0.36
Job strain	**−0.12**	**2.15**	**0.04**	**− 0.12**	**2.16**	**0.04**
Support at work	0.03	0.13	0.63	0.03	0.13	0.64
Physical activity	−0.03	0.44	0.62	−0.04	0.45	0.55
Beliefs to be back at the same work	**0.39**	**0.12**	**0.001**	**0.39**	**0.13**	**0.001**
Anxiety	−0.14	0.13	0.07	−0.13	0.13	0.08
Depression	−0.15	0.15	0.07	−0.15	0.15	0.06
Age				−0.09	0.04	0.13
Economic situation				−0.04	0.45	0.63
*R square*	***0.52***			***0.53***		
*Adjusted R* ^*2*^	***0.49***			***0.48***		

*β* Standardized regression coefficient, *SE* Standard Error

Note: Bold numbers represent significant values (*p* < 0.05)

Table 4 shows that women with higher self-efficacy had greater well-being (β = 0.14, *p* < 0.05). As the women’s scores for depression increased, their well-being decreased by 48% (β = − 0.48, *p* < 0.001). Economic situation *per se* was significantly related to well-being (β = 0.14, *p* < 0 .05). The regression model was significant (*p* < 0.001) and explained 59% of total the variation in well-being.

**Table 4:** Multiple linear regression analyses between the independent variables and well-being

Variables	Well-being (*n* = 168)					
	Unadjusted analysis			Adjusted analysis		
	β	SE	*p*-value	β	SE	*p*-value
Pain intensity	0.05	0.55	0.45	0.06	0.57	0.36
Self-efficacy	**0.16**	**0.12**	**0.02**	**0.14**	**0.12**	**0.03**
Sense of coherence	**0.17**	**0.06**	**0.04**	0.14	0.06	0.09
Divert attention	−0.09	0.12	0.19	−0.10	0.13	0.16
Ignore sensation	−0.04	0.11	0.52	−0.02	0.11	0.82
Increase behavioral activities	0.02	0.13	0.80	−0.01	0.13	0.91
Job strain	0.08	2.60	0.17	0.08	2.58	0.15
Support at work	−0.09	0.16	0.15	−0.09	0.16	0.17
Physical activity	0.03	0.53	0.60	0.03	0.53	0.65
Beliefs to be back at the same work	0.02	0.14	0.78	0.01	0.15	0.96
Anxiety	−0.08	0.16	0.33	−0.08	0.16	0.30
Depression	**−0.49**	**0.18**	**0.001**	**−0.48**	**0.18**	**0.001**
Age				−0.03	0.05	0.57
Economic situation				**0.14**	**0.54**	**0.02**
*R square*	***0.62***			***0.63***		
*Adjusted R* ^*2*^	***0.59***			***0.59***		

*β* Standardized regression coefficient, *SE* Standard Error

Note: Bold numbers represent significant values (*p* < 0.05)

## Discussion

The present findings revealed that women who more strongly believed they would return to the same work had greater work ability, whereas women with higher pain intensity and higher job strain had lower work ability. Women with higher self-efficacy rated greater well-being, and women’s well-being increased as their depression decreased.

### Work ability

In the present study, one of the important factors for women’s work ability was pain intensity. Our finding that higher pain intensity is associated with reduced work ability is not surprising and consistent with results from previous studies showing that increased MSP is independently associated with lower work ability in female laboratory technicians as well as young and old workers [5, 13]. Other studies have found an impact of high intensity of MSP including neck/shoulder and back, on low work ability among women and men [41, 42].

We also found that high-job strain, i.e., high demands in combination with low decision latitude, was related to reduced work ability among women on long-term sick leave due to pain in the neck/shoulders and/or back. The result is in accordance with previous findings suggesting that high-job strain causes poor work ability [43]. Further, studies also found job strain to be an indicator of increased risk of long-term sick leave and MSP intensity [44, 45].

Our results also showed that women who believed they would return to the same work rated greater work ability. This is in line with a cross-sectional study demonstrating that internal health-related control beliefs are an important individual resource that might moderate the effect of work-related stressors on work ability [46]. A prospective cohort study also found recovery beliefs to be a predictor of return to work among male and female workers with chronic low-back pain who were receiving sickness benefits [15].

Contrary to our expectations, self-efficacy and sense of coherence did not significantly contribute to work ability. This was surprising, as they have been found to be significant in previous studies among whiplash and chronic and sub-acute MSP patients [21, 22]. Similarly, factors such as coping strategies, i.e., divert attention, ignore sensation and increase behavioral activities, and support at work did not appear to be significant in the present study, but have been found to be significant in previous studies for work ability [19, 47]. One possible explanation is that the participants in our study were women on sick leave because support at work is likely of more importance to people who are working.

### Well-being

The present study indicated that high self-efficacy was associated with increased well-being. The finding is supported by previous studies suggesting that self-efficacy is an important coping factor among chronic pain patients [48, 49]. As expected, our study revealed an inverse association between depression and well-being, suggesting that depression decreases well-being. This was not the case for anxiety. Possibly, anxiety is obscured by the high prevalence of depression in this population [50].

In our study, different factors were identified for work ability and well-being, respectively, meaning that different factors need to be targeted to achieve greater work ability as opposed to greater well-being. For example, the factors self-efficacy and depression were found to be significant for well-being, whereas these factors were not significantly related to work ability. It should be noted, however, that the factors were close to significant for work ability. Previous studies have found self-efficacy and depression to be important for work ability in patients with chronic whiplash-associated disorder and for employees on long-term sick leave [14, 21]. Our adjusted analysis showed that the confounding factor, economic situation, itself was significantly associated with well-being. If we regard economic situation as a personal factor, it is reasonable to assume when personal economy is good, it could provide access to other coping resources that support improved well-being [51–53].

### Strengths and limitations

One of the strengths of the present study is the use of validated scales to measure all independent variables and outcomes and use of well-defined inclusion criteria. The selection of participants was based solely on what was specified in the medical certificate issued by physician. Thus, participants’ own opinions of their illness were not considered. Further, pain figure was used in order to check with the information about pain in the neck/shoulders and/or back. In the invitation letter sent to the participants, it was stressed that the project was made in collaboration between the SIA and the University of Gävle, and that non-response would not affect the women’s right to compensation. We believe that our results were not affected by SIA selecting participants. Two of the authors (MLK, AN) instructed personnel at SIA in how to select participants based on the inclusion and exclusion criteria.

The study has some limitations that should be noted. The number of non-response could raise questions about potential selection bias. We believe there are two major reasons for the low response rate: (i) participation was voluntary and no compensation was offered, and (ii) participants were unable to answer all the questions due to pain. To increase the response rate, we sent two consecutive reminders. As the study was cross-sectional in design, inferences about cause and effect cannot be made. Longitudinal studies are required to confirm these results. Job strain and support at work, as measured by the DCSQ, showed low internal consistency. Because work ability and well-being were measured using self-reported data, it is possible that the results would have been different if objective measurements had been applied.

## Conclusion

The present findings suggest that beliefs to be back at the same work, pain intensity and job strain are predictors of work ability, while self-efficacy and depression are predictive of well-being among women on sick leave due to long-term pain in neck/shoulders and/or back. Hence, the present study showed that the factors associated with work ability were not the same as those associated with well-being in this population. Given the differences between the outcomes, we believe it is important to consider work ability as well as well-being in the population. The findings highlight factors that should be considered by health care professionals and policy-makers to guide attempts to reduce sick leave. The results may not be generalizable to men, as gender may modify the examined relations.

## Abbreviations

CSQ: Coping Strategies Questionnaire
DCSQ: Demand Control Support Questionnaire
GSE: General Self-Efficacy
HADS: Hospital Anxiety and Depression Scale
LiSat: Life Satisfaction
MPI: Multidimensional Pain Inventory
MSP: Musculoskeletal pain
SOC: Sense of Coherence
VIF: Variance Inflation Factor
WAI: Work Ability Index
